# Plant derived extract loaded nanostructured lipid carriers with enhanced oral delivery for the treatment of acute inflammation

**DOI:** 10.1038/s41598-025-17898-y

**Published:** 2025-10-03

**Authors:** Eun Yong Lee, JeongUn Choi, Young Min Kim, Kyungjik Yang, A-yeong Jang, Hae Kyung Shin, Hae Rim Kim, Keonwook Nam, Jae Kwan Hwang, Jiyong Park, Woo Jung Park, Young Hoon Roh

**Affiliations:** 1 Graduate Program in Bioindustrial Engineering, College of Life Science and Biotechnology, Yonsei University, 50 Yonsei-ro, Seodaemun-gu, Seoul, 03722 Republic of Korea; 2 Department of Marine Food Science and Technology, Gangneung-Wonju National University, Gangneung, 25457 Republic of Korea; 3 Department of Biotechnology, College of Life Science and Biotechnology, Yonsei University, 50 Yonsei-ro, Seodaemun-gu, Seoul, 03722 Republic of Korea; 4 Nutrex Technology, 43 Changeop-ro A-801, Seongnam, 13449 Republic of Korea; 5 Department of Marine Bio Food Science, Gangneung-Wonju National University, Gangneung, 25457 Republic of Korea

**Keywords:** Xanthorrhizol, Curcumin, Nanostructured lipid carriers, Oral delivery, Anti-inflammatory effect, Biotechnology, Nanoscience and technology

## Abstract

Xanthorrhizol and curcumin are bioactive compounds derived from plants, including *Curcuma xanthorrhiza*, a member of the ginger family. However, their limited solubility leads to low oral bioavailability and poses significant constraints for industrial applications. Therefore, we developed nanostructured lipid carriers (NLCs) loaded with standardized ethanolic *Curcuma xanthorrhiza* extract (CXE). The CXE-NLCs were optimized by varying the initial concentration of CXE, resulting in homogeneous particles with a size of 130.0 ± 1.0 nm and a high encapsulation efficiency of approximately 83%. CXE-NLCs exhibited enhanced biostability under simulated gastrointestinal conditions, and in vitro release studies demonstrated the sustained release of CXE. Additionally, CXE-NLCs demonstrated significantly improved permeability across the Caco-2/HT-29 cell monolayer (P_app_ = 4.2 × 10^− 5^ cm/s), which is favorable for the oral delivery of bioactive compounds. In vivo studies confirmed that CXE-NLCs inhibited carrageenan-induced inflammation in the paw, prostaglandins E_1_ and E_2_, as well as the eicosanoid and MAPK pathways in a dose-dependent manner. This study highlights the enhanced oral delivery and bioactivity of synthesized CXE-NLCs containing both xanthorrhizol and curcumin in the treatment of acute inflammation. This indicates a useful application and suggests their potential as a nano delivery system for nutraceuticals and pharmaceuticals.

**Supplementary Information:**

The online version contains supplementary material available at 10.1038/s41598-025-17898-y.

## Introduction

Xanthorrhizol (Xan), a bisabolene-type sesquiterpenoid, and curcumin (Cur), a natural polyphenolic compound, are potent bioactive compounds with anti-inflammatory, anticancer, antimicrobial, and antioxidant activities^1–3^. *Curcuma xanthorrhiza*, also known as Java turmeric, belongs to the ginger family. Its rhizome contains various sesquiterpenes (xanthorrhizol, bisacumol, bisacurol, bisacurone, and zingiberene) and curcuminoids (curcumin, mono-demethoxycurcumin, bisdemethoxycurcumin, etc.)^4^. The ethanol extraction process for Java turmeric is a cost-effective method that utilizes a simple heating process for edible materials. It yields high concentrations of bioactive compounds, particularly Cur and Xan, thereby enhancing their bioactivity^5^. However, the practical application of *Curcuma xanthorrhiza* extract (CXE) is challenging because of its immiscibility in aqueous solutions, instability in the gastrointestinal tract, rapid systemic elimination, low intestinal permeability, and limited bioavailability. Nano- and microscale delivery systems for CXE have been investigated to address these challenges. These include chitosan-TPP-based nanosuspensions and spray-dried microcapsules^6,7^. However, the oral delivery of CXE remains largely understudied despite its therapeutic potential. This highlights the need for an effective delivery system that can overcome the poor solubility, instability in the gastrointestinal tract, and limited absorption of this extract.

Bioactive compounds, such as phytochemicals (e.g., quercetin, curcumin, β-carotene, and α-tocopherol), vitamins (e.g., ascorbic acid, vitamin E, and vitamin D3), functional lipids, proteins/peptides (e.g., insulin and collagen), and nucleic acids (e.g., ODN, siRNA, and mRNA), are combined with suitable colloidal particles to address their limited solubility in physiological fluids and their susceptibility to light, chemical, and biological degradation^8–10^. Lipid-based nanoparticles have gained tremendous attention for increasing the stability and bioavailability of hydrophobic bioactive compounds owing to their biocompatibility, high drug-loading efficiency, and better intracellular penetration. In terms of structure and crystallinity, lipid-based nanoparticles are categorized into nanostructured lipid carriers (NLC) and solid lipid nanoparticles (SLN). Although SLNs offer benefits such as biodegradability and protection of hydrophobic drugs, their applicability is often limited by their highly ordered crystalline structure. This structure results from the exclusive use of solid lipids, which restricts drug loading and promotes drug expulsion during storage^11^. NLCs have been used to improve the bioavailability and physicochemical stability of various bioactive compounds, including curcuminoids, lipophilic vitamins, carotenoids, and flavonoids^12–14^. NLCs consist of a combination of liquid and solid lipids in specific ratios; the liquid lipid enhances loading capacity, whereas the solid lipid prevents agglomeration and/or flocculation. The encapsulation of hydrophobic compounds in NLC leads to improved bioaccessibility, including increased apparent permeability, sustained release of cargo, improved stability in the gastrointestinal tract, and enhanced oral bioavailability^12^. These benefits stem from the partially crystallized lipid matrix formed by mixing solid and liquid lipids, which introduces structural imperfections that increase drug loading and reduce drug expulsion during storage^11^. Conventional lipid-based delivery systems often exhibit instability, premature leakage, and limited control over drug release. Contrastingly, NLCs offer a robust platform that protects encapsulated bioactive compounds and facilitates targeted delivery to intestinal absorption sites.

Inflammation is an essential defense process that protects organisms from physical, chemical, and infectious threats. However, the inflammatory response to various stimuli may inadvertently damage healthy tissues. Acute inflammation is typically a short-term reaction to tissue damage that involves increased blood flow, temperature, swelling, and pain^15^. During acute inflammation, soluble mediators such as cytokines, chemokines, and acute phase proteins can induce the expression of anti-inflammatory mediators, such as prostaglandins (PGs) that promote the resolution of inflammation^16^. In addition, recent research has shown that mitogen-activated kinases (MAPKs) are cell signaling proteins implicated in inflammation and are essential for acute macrophage inflammatory responses^16,17^. CXE and its major components regulate the MAPK pathway and reduce the inflammatory effects of various sources such as lipopolysaccharides, dextran sulfate, 12-O-tetradecanoylphorbol-13-acetate, and carrageenan^17–19^. The bioactivities of CXE, such as its anti-inflammatory effects, have been studied[^20^]. However, its encapsulation and emulsification for efficient oral delivery in nanoprecision have not been studied in vivo.

In this study, we synthesized CXE-loaded NLCs (CXE-NLCs) and evaluated their colloidal stability, bioactivity, and anti-inflammatory effects. We hypothesized that the encapsulation of hydrophobic CXE with robust NLC could improve its miscibility in aqueous solutions, colloidal stability in the gastrointestinal tract, and bioaccessibility in physiological systems. The CXE-NLC was optimized by assessing its loading efficiency, particle size, dispersity, and zeta potential. Its morphological and thermal properties were also characterized. For oral delivery, we examined the gastrointestinal stability, in vitro release profiles, and apparent permeability coefficients of the bioactive compounds. The in vivo anti-inflammatory effects were validated by evaluating the expression levels of inflammatory markers based on paw size, ELISA, and western blotting.

## Materials and methods

### Materials

Glyceryl tristearate, soybean oil, Tween 80, Span 80, and curcumin (≥ 65%) were procured from Sigma-Aldrich (St. Louis, MO, USA). Xanthorrhizol (≥ 99.5%) was obtained from Santa Cruz Biotechnology (Dallas, TX, USA). Heat-inactivated fetal bovine serum (FBS) and streptomycin were purchased from Corning (Corning, NY, USA). All remaining reagents were purchased from Sigma-Aldrich. Analytical grade solvents and reagents were used in the analysis.

### Preparation of standardized CXE

**Fig. 1:**
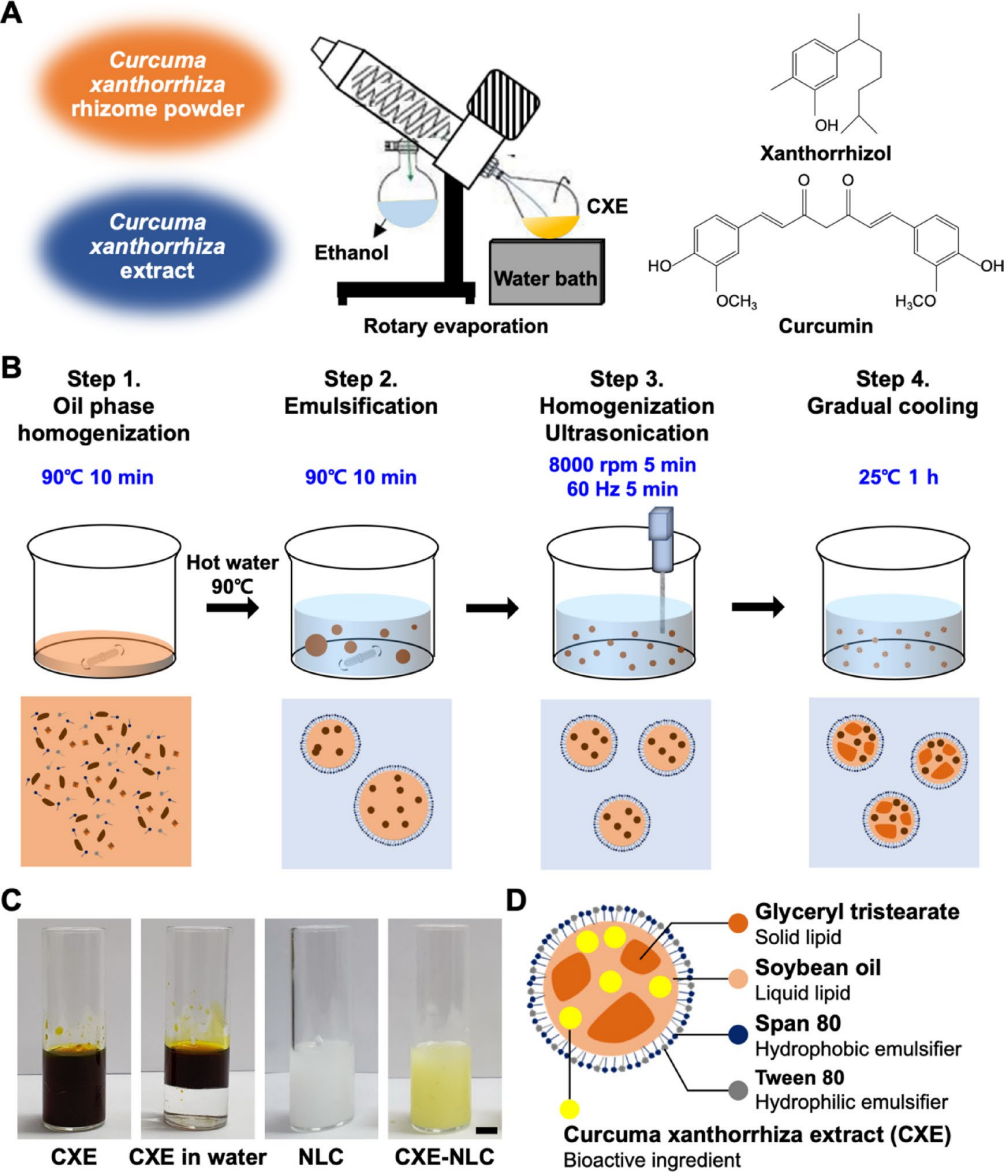
Schematic representation of the fabrication process for CXE-NLC. (**A**) Scheme for standardized CXE preparation from CX rhizome powder. (**B**) Scheme for the synthesis of CXE-NLC. (**C**) Digital photographs of CXE, CXE in water, blank NLC, and CXE-NLC. Scale bar: 1 cm. (**D**) Graphical illustration of CXE-NLC components.

*Curcuma xanthorrhiza* (CX) was collected from Jakarta, Indonesia, using a voucher specimen provided by the Department of Biotechnology, Yonsei University (Seoul, South Korea). The standardized extract was prepared as previously described^5^, with slight modifications. To summarize, 100 g of dried CX rhizome powder was extracted with a 95% ethanol solution for 3 h at 60 °C. The resulting filtrate was evaporated using a rotary evaporator (Heidolph Instruments GmbH & Co. KG, Schwabach, Germany) (Fig. 1A). CXE was standardized based on Xan and Cur contents, which were quantified using high-performance liquid chromatography (HPLC). The HPLC conditions and standard plots for Xan and Cur are described in the Supplementary Data (Fig. S1 and Fig. S2). CXE analysis was performed using reverse-phase HPLC (YL3000; YoungLin Instrument, Anyang, South Korea) with a C18 column (ZORBAX Eclipse Plus, 4.6 × 250 mm, 5 μm particle size; Agilent Technologies, Santa Clara, CA, USA). The HPLC conditions included a total analysis time of 90 min, flow rate of 1 mL/min, a linear solvent gradient (0 min: 45% acetonitrile (ACN), 55% 0.01% formic acid (FA); 60 min: 85% ACN, 15% FA; 75 min: 100% ACN; 80 min: 45% ACN, 55% FA; 90 min: 45% ACN, 55% FA), isothermal column oven temperature of 30 °C, and absorbance detection at 229 nm. Before injection, CXE was diluted 100-fold with ethanol, and 10 µL of the diluted sample was injected for analysis.

### Preparation of CXE-loaded NLCs

CXE-NLCs were prepared using high-shear homogenization and ultrasonication techniques. The NLCs were formulated with fixed amounts of glyceryl tristearate (GTS) as the solid lipid, soybean oil as the liquid lipid, and Tween 80 and Span 80 as the hydrophilic and hydrophobic surfactants at 600, 240, 400, and 270 mg, respectively, to synthesize 100 mL of NLC. CXE was used at concentrations of 0.3, 0.5, 1, 2, and 4 g per 100 mL of NLC, resulting in final concentrations of 0.3, 0.5, 1.0, 2.0, and 4.0% (w/v). Briefly, the oil phase was prepared by mixing GTS, soybean oil, Tween 80, Span 80, and CXE and heating to 90 °C while stirring. Once the lipids melted, 100 mL of distilled water heated to the same temperature (water phase) was added dropwise, and the solution was stirred at 450 rpm for 10 min. This pre-emulsion was then subjected to high-shear homogenization (HZ1; LABTron Co., South Korea) at 8,000 rpm for 5 min, followed by further dispersion using a probe sonicator (UP200S; Hielscher Ultrasonics GmbH, Germany) for an additional 5 min (amplitude power: 30%; work time: 4 s; rest time: 2 s). Finally, the CXE-NLCs were gradually cooled to 25 °C to allow crystallization of the solid lipid. Blank NLCs were synthesized without CXE using the same fabrication process.

### Physicochemical characterization of CXE-NLCs

The particle size, polydispersity index (PDI), and zeta potential of the CXE-NLCs and blank NLC were determined through dynamic light scattering (DLS) and electrophoretic light scattering (ELS) using a particle size and zeta potential analyzer (ELS-2000ZS; Otsuka Electronics Co., Osaka, Japan). In order to obtain a fourier transformation infrared (FT-IR) spectra, CXE was lyophilized with hydroxy propyl methyl cellulose (HPMC), and blank-NLC and CXE-NLC were also lyophilized. The FT-IR spectra of CXE-HPMC, HPMC, GTS, blank-NLC, and CXE-NLC were obtained using a VERTEX 70 FT-IR spectrometer (Bruker, Billerica, MA, USA) in the ranges of 4,000 ~ 400 cm-1. Differential scanning calorimetry (DSC) and thermogravimetric analysis (TGA) were performed on the CXE, blank NLC, and CXE-NLC using a thermal analyzer (SDT Q600; TA Instruments, DE, USA). The temperature range was 25–600 °C, with a heating rate of 20 °C/min under a nitrogen gas flow of 100 mL/min. Field emission scanning electron microscopy (FE-SEM; JSM-7001 F; JEOL, Tokyo, Japan) was used to visualize the morphology of the CXE-NLCs. The CXE-NLCs were dried overnight on a silicon wafer and observed at an accelerating voltage of 5 kV. Transmission electron microscopy (TEM; JEM-F200; JEOL, JAPAN) was used to examine the crystalline structure of the CXE-NLCs at an acceleration voltage of 200 kV. The samples were diluted 20-fold and loaded onto a carbon/formvar copper grid (300 mesh; Electron Microscopy Sciences, Hatfield, PA, USA). The particle sizes in the SEM and TEM images were measured using ImageJ software (v1.8, National Institutes of Health, Bethesda, MD, USA). The diameters of 50 particles were measured, and the average values and standard deviations were calculated. The colloidal stability of the CXE-NLC was evaluated at 4 °C for six weeks, with its particle size, PDI, and zeta potential monitored throughout.

### CXE loading efficiency and capacity

The encapsulation efficiency (EE) and loading capacity (LC) of CXE-NLCs were determined using the ultrafiltration-centrifugation technique as previously described with slight modifications^21^. An Amicon centrifugal filter device (MWCO 10,000 Da; Millipore, Billerica, MA, USA) was used to remove unencapsulated CXE during centrifugation at 12,000 × *g* for 80 min. The amount of unencapsulated CXE in the NLC was analyzed by measuring fluorescence intensity. A standard curve was generated using CXE (Fig. S3), and EE and LC were calculated using the following equations:

EE (%) = [(Total weight of added ingredient – Weight of unentrapped ingredient) / Total weight of added ingredient] × 100.

LC (%) = [(Total weight of added ingredient – Weight of unentrapped ingredient) / (Total weight of added ingredient – Weight of unentrapped ingredient + Weight of lipid phase in the nanoparticle formulation)] × 100.

### In vitro intestinal permeability

The apparent permeability coefficients (P_app_, cm/s) were measured using a Caco-2/HT-29 co-cultured cell monolayer as previously described^10^. Caco-2 and HT-29 cells were obtained from the Korean Cell Line Bank (Seoul, South Korea) and cultured in minimum essential medium (MEM) and McCoy’s medium, respectively. Cells were seeded at a ratio of 9:1 on Transwell inserts (SPL Insert™ Hanging; SPL Life Science, Pocheon, South Korea) at a density of 1 × 10^5^ cells/well. The growth medium was replaced every 2–3 days with Dulbecco’s modified Eagle’s medium (DMEM) supplemented with 1% penicillin/streptomycin and 10% FBS. The cells were then incubated at 37 °C in 5% CO_2_. Transepithelial electrical resistance (TEER) was measured using an EVOM2 Epithelial Volt/Ohm Meter (World Precision Instruments, Sarasota, FL, USA) to confirm formation of the co-culture monolayer. Cell monolayers were used when the TEER values ranged from 400 to 600 Ω∙cm^2^. After confirming cell monolayer formation, the cells were washed with PBS and equilibrated with Hank’s balanced salt solution (HBSS) transport buffer. Subsequently, 200 µL of HBSS buffer containing CXE or CXE-NLC with the same amount of CXE (at a dose of 125 µg/mL) was applied to the cells. Before evaluation, CXE and CXE-NLCs were treated with enzyme-free simulated gastric fluid (SGF) and simulated intestinal fluid (SIF) for 2 h. Samples were collected after 1 h and the amount of transported CXE was determined by measuring the fluorescence intensity. The P_app_ of the treated samples were calculated using the following equation:

P_app_ = dQ/dt × 1/(A × C_0_).

where Q, A, C_0_, and t represent the amount of CXE transported during treatment (µg), surface area of the cell monolayer (cm^2^), initial amount of CXE in the donor chamber (µg/cm^3^), and total treatment time (s) respectively.

### In vitro biostability in simulated gastrointestinal medium

The stability of CXE-NLCs in SGF and SIF was assessed by measuring the changes in particle size following incubation. SGF and SIF were prepared as previously described (Minekus et al., 2014), with slight modifications. SGF (pH 1.2) containing pepsin (2,000 U/mL) was prepared using 0.7% (v/v) hydrochloric acid and 0.2% (w/v) sodium chloride. SIF (pH 6.8) containing pancreatin (20% w/v) and 10 mM bile salt was prepared using 0.9% (w/v) sodium hydroxide and 6.8% (w/v) monobasic potassium phosphate. Subsequently, the CXE-NLCs were incubated in SGF and SIF at 37 °C for 2 h to simulate the digestive environment. The NLC particle size was determined using DLS before and after exposure to SGF and SIF.

### In vitro release profile of bioactive compounds

The release pattern of CXE from the CXE-NLCs under simulated digestive conditions was assessed using a dialysis membrane (MWCO, 3,500 Da; Thermo Fisher Scientific, Waltham, MA, USA). The membranes were filled with CXE-NLCs and an enzyme-free digestive medium. To prevent leakage, both ends of the membrane were securely tied and immersed in a mixture of enzyme-free SGF and SIF containing 50% ethanol (v/v) to maintain sink conditions. The CXE-NLC-filled membranes were then incubated at 37 °C and 90 rpm sequentially in SGF for 2 h and SIF for 6 h. Samples were collected at specific intervals throughout the incubation process, and the amount of CXE released from the CXE-NLCs was determined by measuring fluorescence intensity.

### In vivo experiments

All animal experiments were approved by the Institutional Animal Care and Use Committee (IACUC) of Gangneung-Wonju National University (approval number: GWNU-2018-20) and were conducted in accordance with relevant guidelines and regulations, including the ARRIVE guidelines (https://arriveguidelines.org/). Male ICR mice (25 ± 2 g; KOATECH, Inc., Pyeongtaek, South Korea) were housed in a facility with controlled temperatures (22 ± 2 ℃) and humidity (55 ± 10%), maintained on a 12 h light/dark cycle, and provided with ad libitum access to food and water throughout the study period. To ensure animal welfare, the animals were monitored daily for signs of distress or abnormal behavior. Humane endpoints were predefined, and animals showing severe signs of pain, distress, or loss of more than 20% of their baseline body weight were to be euthanized immediately. None of the animals met these criteria during the study period. Euthanasia was performed by gradual-fill CO_2_ inhalation in accordance with the American Veterinary Medical Association (AVMA) Guidelines for Euthanasia of Animals (2020), and confirmed by the absence of respiration and reflexes; cervical dislocation was not performed to avoid unnecessary stress.

### In vivo anti-inflammation effects

The mice were housed under controlled conditions and provided with a standard laboratory diet and water for two weeks before commencing the experiment. Mice were randomly divided into eight groups (*n* = 5 per group): normal, carrageenan, L-glutamine (250 mg/kg BW), CXE (200 mg/kg BW), blank NLC (0 mg/kg BW), low (50 mg/kg BW), medium (100 mg/kg BW), and high (200 mg/kg BW) doses of CXE-NLCs. Mice in the non-treated and control groups were orally administered 0.9% saline solution, whereas the other groups were orally administered L-glutamine, CXE, blank NLCs, or different doses of CXE-NLCs in saline solution for 1 day. To induce paw edema in the treatment groups, intraplantar injection of 50 µL of 1% carrageenan was performed in the right hind paw 1 h after oral administration. Paws were measured using a digital caliper before and after 1, 2, 3, 4, and 5 h of carrageenan injection.

### Protein expression analysis in physiological systems

Mice were anesthetized with CO_2_ 5 h after carrageenan injection, and blood samples were collected from the abdominal aorta. Serum was obtained through centrifugation at 1000 × *g* for 20 min at 4 °C and stored at − 20 °C until further analysis. The separated serum was analyzed for PGE1 (PGE1; Enzo Life Science, Farmingdale, NY, USA) and PGE2 (Enzo Life Science) using ELISA kits according to the manufacturer’s instructions. Results were analyzed using a standard curve. Subsequently, the mice were euthanized through continued CO_2_ exposure, and the right paw of each group was removed for protein extraction. Paw tissues were homogenized using a homogenizer and proteins were extracted using RIPA buffer (Tech & Innovation, Hebei, China). Next, protein concentrations were determined using the Pierce™ BCA Protein Assay Kit (Thermo Fisher Scientific). Equal amounts of proteins were separated via sodium dodecyl sulfate-polyacrylamide gel electrophoresis (SDS-PAGE) and transferred to polyvinylidene fluoride (PVDF) membranes. The membranes were then incubated with primary antibodies specific to the following transcription factors: p-p38, p-ERK, p-JNK, COX-1, and COX-2 (Cell Signaling Technology, Danvers, MA, USA), as well as cytosolic phospholipase A_2_ (cPLA2) and α-tubulin (Abcam, Cambridge, UK). The membranes were then washed, and the blots were incubated with Goat Anti-Rabbit IgG(H + L)-HRP (GenDEPOT, Katy, TX, USA) at 37 °C for 1 h. The bands were visualized using Pierce^®^ ECL Plus Western Blotting Substrate (Thermo Fisher Scientific), the ChemiDoc XRS + imaging system, and ImageLab software (Bio-Rad, Hercules, CA, USA).

### Statistical analysis

In vitro experiments were conducted three times, and the data are presented as the mean ± standard deviation (SD). Group comparisons were evaluated using analysis of variance (ANOVA), followed by the Scheffe and Dunnett T3 tests, using SPSS Statistics for Windows (Version 24.0; IBM, Armonk, NY, USA). For the in vivo results, ANOVA was employed, and post-hoc analysis was performed using Duncan’s multiple range test at a significance level of *p* < 0.05.

## Results and discussion

### Synthesis of CXE-loaded NLC

CXE-NLCs were synthesized using a precisely controlled approach to overcome the immiscibility of CXE in the aqueous phase. The carriers were formulated with edible lipids, including GTS, soybean oil, Tween 80, and Span 80. The types and ratios of the lipid components were carefully selected to meet the encapsulation efficiency and stability requirements. Due to the lipophilic nature of CXE, liquid lipid soybean oil is favorable for encapsulating significant amounts of CXE^22^. The solid lipid GTS was incorporated to prevent agglomeration and coagulation by forming stable crystal structures within the nanoparticles. The GTS-to-soybean oil ratio was determined based on previous studies to achieve a balance between encapsulation efficiency and colloidal stability^23^. The process involved several steps. First, the water-immiscible bioactive compound (CXE) was extracted with ethanol using a rotary evaporator. Measurement of CXE composition through reverse-phase HPLC verified that CXE was a mixture of various phytochemicals, such as the curcuminoid curcumin and sesquiterpenes such as xanthorrhizol. CXE was mainly composed of approximately 13.8% Xan and 0.4% Cur (w/w) (Fig. S1). Next, the lipids and emulsifiers were melted and stirred gently at 90 °C (Fig. 1B). A small quantity of CXE (0–4%) was added to the lipid mixture, followed by the introduction of preheated water at the same temperature for primary emulsification. To achieve homogeneity, the resulting emulsions were subjected to high-shear homogenization at 8000 rpm for 5 min, followed by ultrasonication for 5 min. Finally, the emulsion solution was allowed to stand at 25 °C for gradual cooling and crystallization of GTS, which enhanced colloidal stability. Contrary to the insoluble CXE solution demonstrated by the phase separation depicted in Fig. 1C and D, the resulting CXE-NLCs appeared as a yellow homogeneous solution, indicating the successful incorporation of CXE. Conversely, blank NLC fabricated without CXE formed a homogeneous opaque white solution. This nanoformulation overcomes the limitations associated with low CXE solubility and oral bioavailability in industrial food applications.

### Optimization of CXE-loaded NLC

**Fig. 2:**
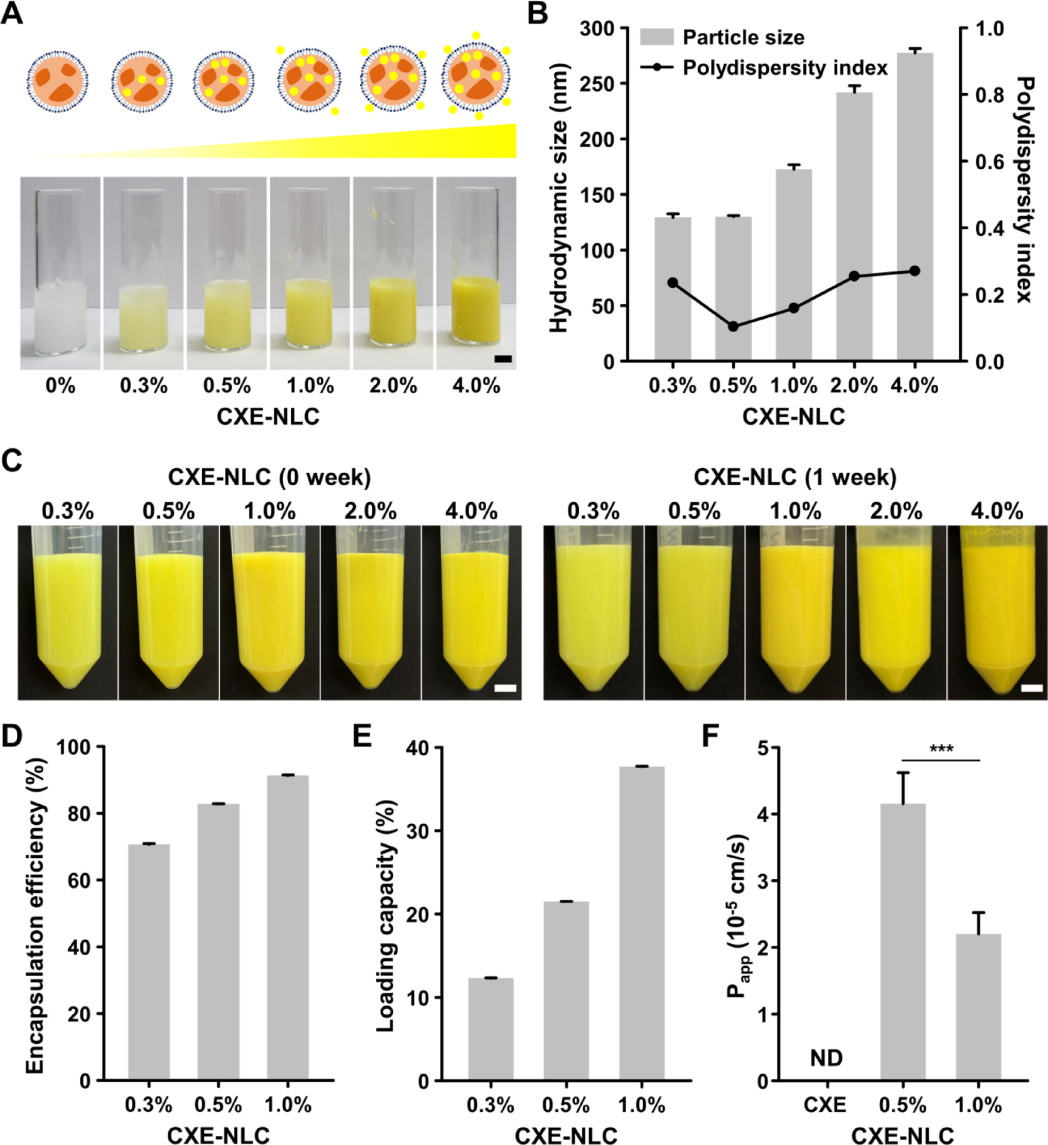
Synthesis and characterization of CXE-NLCs at different initial CXE concentrations. (**A**) Digital images of CXE-NLCs with varying CXE concentrations (0.0–4.0%, w/v). Scale bar: 1 cm. (**B**) Particle size and polydispersity index (PDI) of CXE-NLCs with varying CXE concentrations (0.3–4.0%, w/v). (**C**) Colloidal stability of CXE-NLCs with varying CXE concentrations (0.3–4.0%, w/v), as determined via digital images captured at the time of fabrication and subsequently after one week of storage (4 °C). (**D**) Encapsulation efficiency of CXE-NLCs (0.3–1.0%, w/v). Scale bars: 1 cm. (**E**) Loading capacity of CXE-NLCs (0.3–1.0%, w/v). (**F**) Apparent permeability coefficients (P_app_) of CXE (1% DMSO solution) and CXE-NLCs (0.5, 1.0%, w/v) across a Caco-2/HT29 (9:1) cell monolayer. ND: not detected, *** *p* < 0.005.

The EE, LC, hydrodynamic size, and PDI of the CXE-NLC were assessed. CXE-NLCs were successfully fabricated within a CXE concentration range of 0.0–4.0%, without inhomogeneity or phase separation. The formulations were labeled as blank NLC, CXE-NLC-0.3, CXE-NLC-0.5, CXE-NLC-1.0, CXE-NLC-2.0, and CXE-NLC-4.0, corresponding to their initial CXE concentrations (Fig. 2A). Hydrodynamic size measurements showed that concentrations below 1% yielded particles of approximately 200 nm, whereas CXE-NLC-2.0 and CXE-NLC-4.0 exhibited increased sizes of 242 nm and 277 nm, respectively (Fig. 2B). The PDI value of CXE-NLCs was below 0.3, indicating a high degree of nanoparticle uniformity. Particle size is a critical factor in permeation and transport in the small intestine, and particle sizes below 300 nm are advantageous for oral bioavailability^24,25^. However, slight coagulation was observed in CXE-NLC-2.0 and CXE-NLC-4.0 after storage for one week (Fig. 2C). CXE-NLC-2.0 and CXE-NLC-4.0 were excluded from the optimized candidates based on colloidal properties such as colloidal stability. The zeta potential of CXE-NLC-0.3, CXE-NLC-0.5, and CXE-NLC-1.0 after preparation was − 23.5 ± 1.3, -32.2 ± 1.8, and − 24.9 ± 0.9 mV, respectively. This negative surface charge primarily arises from the hydration of the polyethylene glycol (PEG) chains in Tween 80, where the ether oxygen atoms form hydrogen bonds with the surrounding water molecules, consequently inducing a partial negative charge on the nanoparticle surface^26^. Typically, a larger absolute value of zeta potential indicates greater stability, making CXE-NLC-0.5 advantageous for preventing coagulation and enabling long-term storage in the aqueous phase. Figure 2D and **E** show the EE and drug LC of the NLCs as a function of CXE concentration. CXE-NLCs that did not aggregate during storage exhibited significant EE (83% and 91%) and drug LC (21% and 37%) at concentrations of 0.5% and 1.0% CXE.

The intestinal permeability of the bioactive compounds (CXE) was determined using the apparent permeability coefficient (P_app_) measured in an intestinal monolayer. The monolayer consisted of a co-culture of Caco-2 cells for absorption and HT-29 cells for mucus secretion, thereby mimicking an in vivo digestive environment^27^. Intestinal monolayers were treated after sequential incubation of CXE and CXE-NLCs with enzyme-free SGF and SIF for 2 h. The average P_app_ values of CXE-NLC-0.5 and CXE-NLC-1.0 were determined as 4.2 × 10^− 5^ cm/s and 2.2 × 10^− 5^ cm/s, respectively, whereas that of CXE was not measurable (Fig. 2F). The significantly higher P_app_ value of CXE-NLC-0.5 and the immeasurable value of CXE suggest that the optimized NLC formulation can substantially enhance oral bioavailability by improving its solubility. CXE is highly hydrophobic and cannot be absorbed by intestinal monolayers. Various mechanisms contribute to the elimination of NLC systems in vivo. First, the NLC are degraded by intestinal enzymes, leading to the uptake of mixed micelles via the chylomicron pathway. In addition, NLC can be absorbed through intracellular uptake via M cell-to-lymphatic transport, intercellular uptake by enterocytes, and paracellular uptake. Furthermore, CXE released from NLC can be directly absorbed, and NLC can inhibit P-glycoprotein (P-gp) efflux, resulting in increased CXE bioavailability^28^. In summary, the stability of SGF, lipid digestion behavior in SIF, and intestinal permeability suggest that the physical state and composition of NLC protect CXE during digestion and enhance its permeability in the small intestine. Because the aim of this study was to enhance the oral bioavailability of hydrophobic CXE, CXE-NLC-0.5 was selected based on its particle size, uniformity, zeta potential, and intestinal permeability for further validation.

### Physicochemical properties of CXE-loaded NLC

**Fig. 3:**
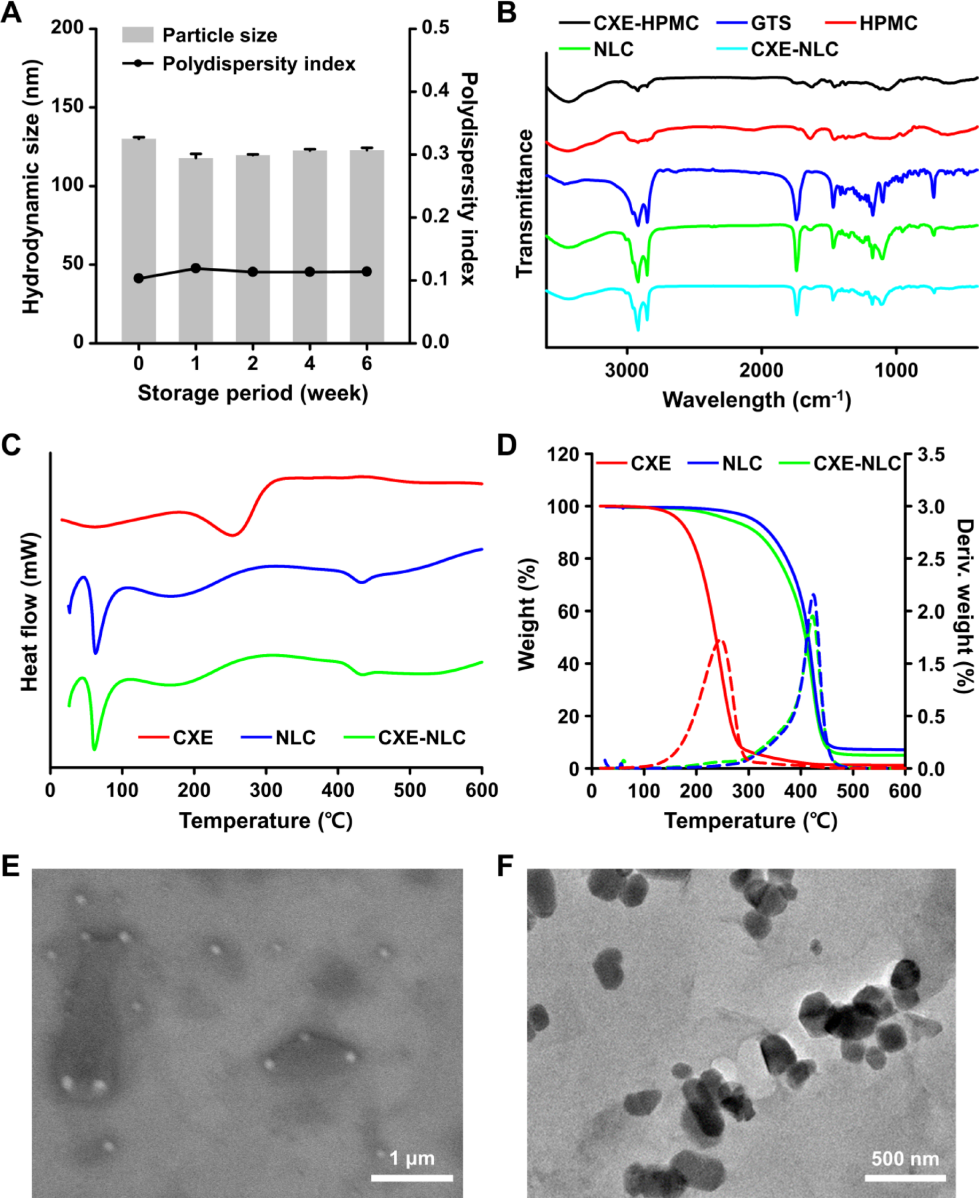
Physicochemical characterization of optimized CXE-NLCs. (**A**) Particle size and PDI of CXE-NLCs during storage periods. (**B**) FT-IR spectra for CXE-HPMC, HPMC, GTS, Blank-NLCs, and CXE-NLCs. (**C**) DSC thermograms of CXE, blank NLCs, and CXE-NLCs. (**D**) TGA curve of CXE, blank NLCs, and CXE-NLCs. Solid and dotted lines indicate changes in weight and derivative weight, respectively. (**E** and **F**) Morphology and structural analyses of CXE-NLCs conducted using (**E**) SEM and (**F**) TEM. Scale bars: 1 μm and 500 nm, respectively.

To assess the colloidal stability of the CXE-NLCs, changes in their physical properties were evaluated under the corresponding conditions. Colloidal stability was determined by incubating CXE-NLCs at 4 °C for 6 weeks and monitoring the agglomeration, hydrodynamic size, PDI, and zeta potential of the particles. CXE-NLC-0.5 showed a highly stable hydrodynamic size and PDI over 6 weeks (Fig. 3A). Furthermore, digital images revealed no visual changes, such as aggregation, creaming, or separation, in CXE-NLC-0.3, CXE-NLC-0.5, and CXE-NLC-1.0, after 4 weeks (Fig. S4). However, visual changes with more aggregation were observed for CXE-NLC-2.0 and CXE-NLC-4.0 during storage. Furthermore, no significant changes in the hydrodynamic size, PDI, and zeta potential of CXE-NLC-0.3, CXE-NLC-0.5, and CXE-NLC-1.0 were observed at 4 °C storage for 6 weeks (Table S1 and Fig. S5). Measurements of particle size and zeta potential for CXE-NLC-2.0 and CXE-NLC-4.0 were not available after 1 week, which was consistent with the digital images that implied that NLC were manufactured with high colloidal stability. The physicochemical properties of the representative CXE-NLC-0.5 were further analyzed using FT-IR spectroscopy, DSC, and TGA. As illustrated in Fig. 3B, the FT-IR spectra of CXE exhibited characteristic absorption bands attributed to O-H absorption at approximately 3400 cm^-1^ and those corresponding to the -CH_3_ and -CH_2_ stretching vibrations at 2800–3000 cm^-1^^29^. The FT-IR spectra of CXE-NLCs revealed no additional peaks compared to those of CXE, blank-NLCs, and GTS. This observation indicated that no new chemical bonds or functional groups were formed during NLC preparation. CXE exhibited a slight exothermic transition from 256 °C in the DSC measurements. This was indicative of glass transition temperature that may be from the extracted amorphous polymeric components, such as carbohydrates. The inherent glass transition of CXE was faint after encapsulation in the NLC, and the heat flow profile was similar to that of the blank NLC (Fig. 3C). The disappearance of the endothermic peak of CXE in the CXE-NLCs indicated that CXE was well-dissolved in the NLC formulation. Analysis of the thermal degradation patterns using TGA demonstrated that the weight mainly decreased around the glass transition temperature in CXE (Fig. 3D). However, the encapsulation process altered the weight loss profile, and CXE-NLCs exhibited a weight loss profile nearly identical to that of the blank NLC.

The morphological and structural properties of CXE-NLCs were further analyzed using SEM and TEM. SEM analysis revealed the formation of monodisperse nanoparticles (Fig. 3E). Because nanoparticle size is critical for intracellular uptake and paracellular transport, particle size was measured (123.9 ± 15.6 nm) using ImageJ. In the TEM images, their particle size was measured as 167.8 ± 37.0 nm (*n* = 50) from ImageJ analysis; this was consistent with the size measured using DLS. In addition, distinct crystalline structures and irregularly faceted or polyhedral morphologies were observed (Fig. 3F). In a previous study, NLC synthesized with a solid-to-liquid lipid ratio higher than 1 was reported for nanoparticles with spherical morphology, and a lower solid-to-liquid lipid ratio produced faceted structures^30^. However, the morphologies, sizes, crystalline structures, and types of NLC may not be solely dependent on the solid-liquid ratio. Rather, they could be affected by other variables, including the type of lipids, loading compounds, and synthetic processes. In the present study, CXE-NLCs with a solid-liquid lipid ratio of 2.5 exhibited irregular structural morphologies. We hypothesized that the considerably larger solid-liquid lipid ratio and high-shear homogenization and ultrasonication resulted in a greater multilamellar structure. The increased multilamellarity and highly unordered imperfect crystals of the solid lipid may have contributed to the angular and irregular structure of CXE-NLCs. Overall, physicochemical analyses provided insights into the thermal and structural properties and localization of CXE within the nanoparticles. Moreover, these analyses confirmed the successful encapsulation of CXE in NLC.

### In vitro bioavailability of CXE-loaded NLC

**Fig. 4:**
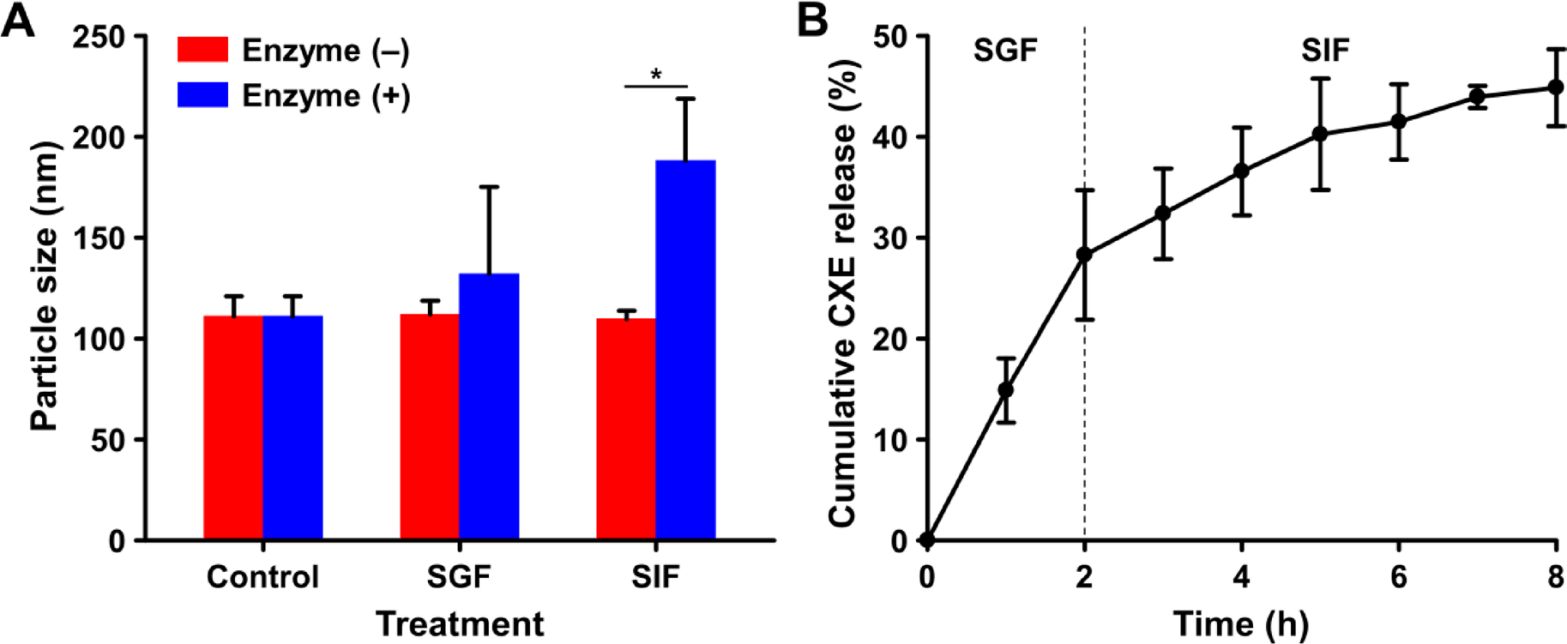
Behavior of CXE-NLC under simulated in vitro gastrointestinal conditions. (**A**) Digestive stability and lipid digestion properties of CXE-NLC in simulated gastrointestinal fluid (stability test conducted without enzyme/digestion assay performed with enzymes). (**B**) In vitro release profile of CXE-NLC in enzyme-free simulated gastrointestinal fluid.

To investigate the digestive stability of CXE-NLCs, these nanocarriers were incubated in SGF with or without enzymes for 2 h, and their hydrodynamic size was monitored (Fig. 4A). No significant changes were observed in the hydrodynamic size of CXE-NLCs incubated in SGF and SIF without enzymes. This indicated that the CXE-NLCs remained stable under physiological pH conditions in the gastrointestinal environment. Subsequently, their hydrodynamic diameter was measured after incubation in SGF containing pepsin and SIF containing lipase and bile salts for 2 h. Digestive enzymes can induce lipid digestion, thereby enhancing the oral bioavailability of CXE-NLCs composed of lipids and surfactants^31^. The hydrodynamic sizes of CXE-NLCs after SGF incubation remained similar, with no signs of aggregation or degradation. The acid stability and pepsin-resistant properties of CXE-NLCs during gastric passage can be attributed to the steric stabilization effect of the nonionic surfactants Tween 80 and Span 80, whose molecular structures prevent aggregation and coalescence^22^. The hydrodynamic diameter of CXE-NLC significantly increased from 107.1 ± 10.8 nm to 171.4 ± 14.7 nm following incubation in SIF. This increase can be explained by the breakdown and aggregation of NLC caused by lipase and bile salts, which leads to the formation of emulsified mixed micelles composed of digested lipids, bile salts, and drugs^13,22^. These micelles are absorbed into the systemic circulation through epithelial cells in the small intestine, consequently facilitating the absorption of bioactive compounds into systemic circulation^32^. Therefore, mixed micelles may contribute to the absorption of NLC-encapsulated hydrophobic CXE bioactive components in the small intestine.

To assess the oral delivery of CXE-NLCs, the in vitro release profile of the nanocarriers were evaluated. The in vitro release profiles of the CXE-NLCs in SGF and SIF without enzymes were evaluated using dialysis (Fig. 4B). CXE-NLCs demonstrated an initial rapid release of CXE in SGF, followed by a gradual sustained release in SIF. Specifically, 28.3% and 16.6% of CXE were released during 2 h of SGF incubation and 6 h of SIF incubation, respectively. The mechanism of CXE release from the NLC involves matrix erosion, diffusion, and release from the nanoparticle surface^33^. The initial rapid release could be attributed to the release of CXE adsorbed near the outer layer of the CXE-NLCs, as no significant change was observed in the hydrodynamic size during SGF incubation. The presence of residual oil on the outer surface of the nanocarriers may contribute to the formation of a soft shell, leading to initial release. The subsequent sustained release in SIF may be due to erosion of the inner hydrophobic lipid matrix of the CXE-NLCs, resulting in slow diffusion of CXE from the NLC^34^. This sustained-release profile increases oral bioavailability and enhances biological activity, owing to prolonged retention in the gastrointestinal tract.

### In vivo anti-inflammatory effect in CXE-loaded NLC

**Fig. 5:**
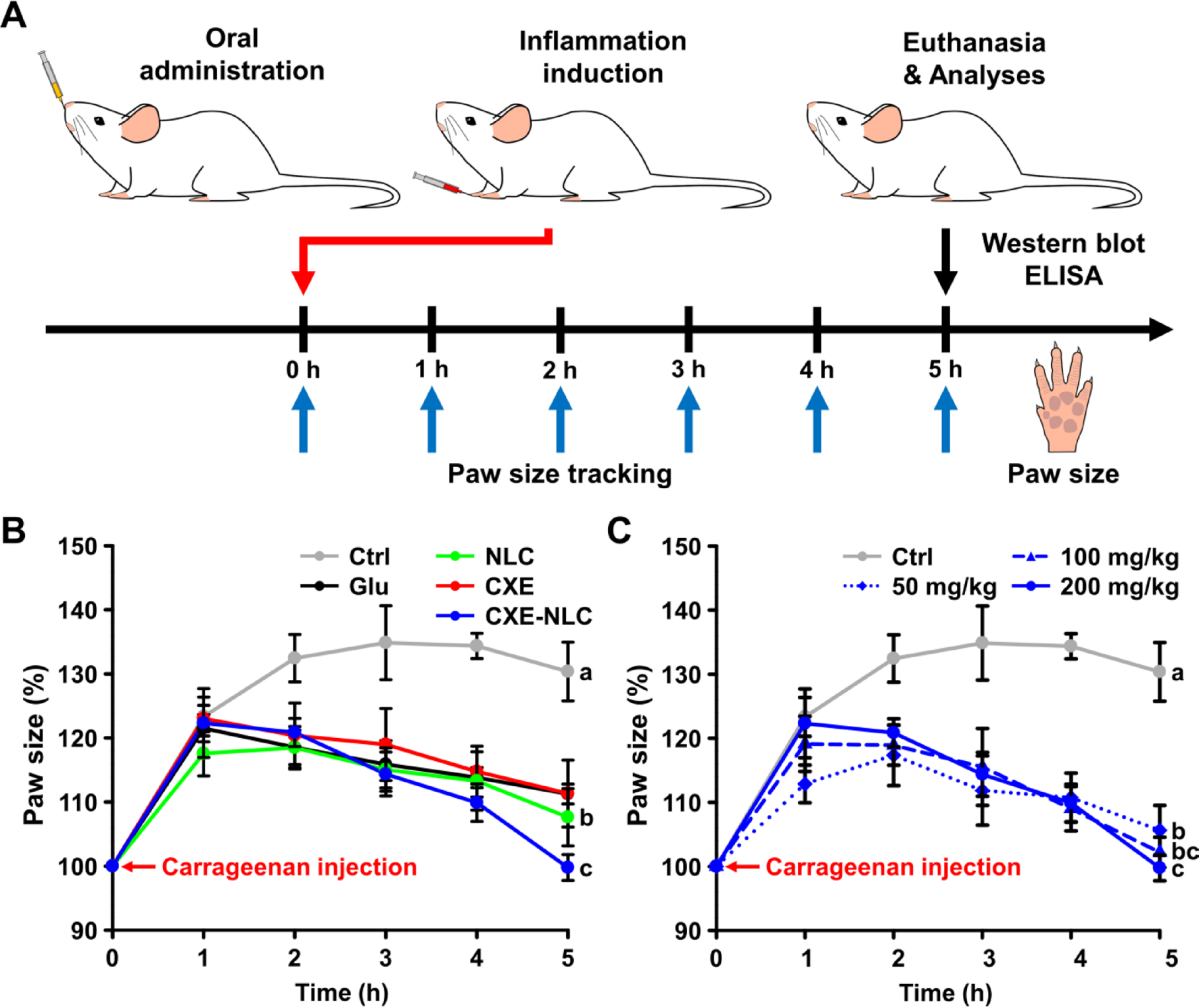
Anti-inflammatory activity of CXE-NLC in mice. (**A**) In vivo experimental design. The initial paw size was measured after oral administration at 1 h. The right hind paw of each mouse was injected with carrageenan to induce inflammation. Subsequently, paw size was assessed at various times over a 5-h period. Blood samples and paw tissues were obtained after 5 h. Western blotting and ELISA were conducted on the blood and paw tissue samples. (**B**) Percentage increase in paw edema following carrageenan injection compared with that in the Control, Glu, NLC, CXE, and CXE-NLC groups. CXE and CXE-NLCs were treated at dosages of CXE 200 mg/kg BW. (**C**) Percentage increase in paw edema following carrageenan injection compared with that in the Control and CXE-NLC groups. Ctrl: carrageenan-treated control; Glu: L-glutamine, 250 mg/kg BW; CXE: free CXE, 200 mg/kg BW; NLC: blank NLC; CXE-NLC: NLCs at dosages of CXE 50, 100, and 200 mg/kg BW. Data are expressed as the mean ± SD (*n* = 5). Statistically significant differences (*p* < 0.05) among all groups are denoted by different letters (a–c).

Injection of carrageenan into mouse paws is a well-established approach for inducing local inflammation, making it suitable for evaluating the efficacy of anti-inflammatory agents^35^. In the present study, we investigated the anti-inflammatory effects of CXE-NLCs on carrageenan-induced paw edema. Figure 5A shows a schematic illustration of the anti-inflammatory mouse model that visually represents key steps of the experimental procedure. The initial paw size was measured, and carrageenan was injected into the right hind paw to induce inflammation 1 h after oral administration of CXE-NLCs. Paw size was subsequently measured at various time points over a 5 h period after induction of inflammation. At 5 h, the paw tissue was dissected, and blood samples were collected. Western blot analysis was performed on proteins extracted from the paw tissue, and ELISA was used to analyze the serum. The results are shown in Fig. 5B and **C**. The intraplantar injection of carrageenan led to progressive paw edema in mice. However, oral administration of CXE-NLCs significantly inhibited paw edema in a concentration-dependent manner at all time points compared with that in the control (carrageenan-treated only) group. CXE-NLCs (200 mg/kg BW) exhibited the most substantial inhibition of paw size. In contrast, the L-glutamine (250 mg/kg BW) and CXE (200 mg/kg BW) groups did not exhibit significant inhibition compared with the CXE-NLCs (50, 100, and 200 mg/kg BW). Calculation of the percentage of paw edema at each time point revealed a suppression of ≥ 10–30% in all treatment groups compared with the control group (carrageenan-treated only). In particular, the paw size of the 200 mg/kg CXE-NLC-treated group showed a significant decrease of approximately 30% compared to that of the control group (carrageenan-treated only). Importantly, CXE-NLCs reduced the inflammatory response more efficiently than the positive control (L-glutamate-treated) and CXE groups, indicating that nano-emulsification strategies using the NLC platform could enhance oral bioavailability and bioactivity.

**Fig. 6:**
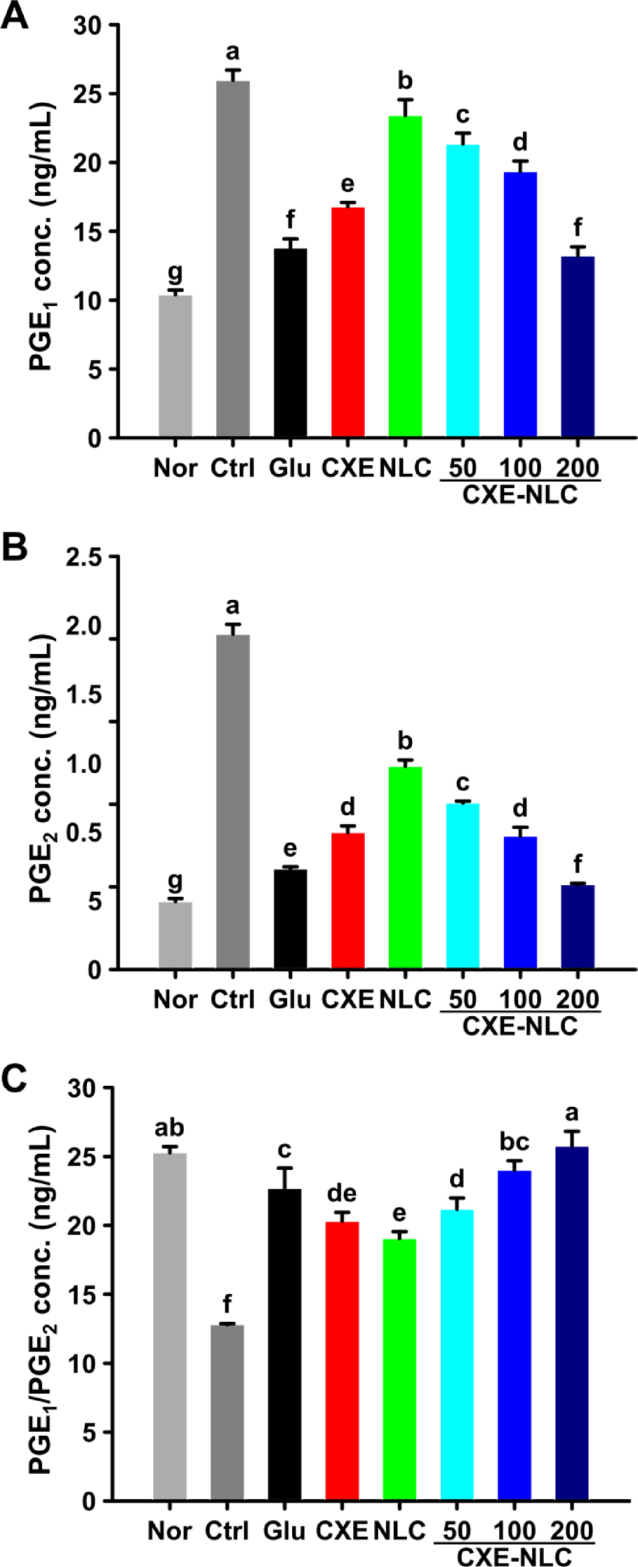
Regulatory effect of CXE-NLC on eicosanoid production. (**A**) PGE_1_, (**B**) PGE_2_, and (**C**) PGE_1_/PGE_2_ ratio. Nor: untreated; Ctrl: carrageenan-treated control; Glu: L-glutamine, 250 mg/kg BW; CXE: free CXE, 200 mg/kg BW; NLC: blank NLC; CXE-NLC: NLCs at dosages of CXE 50, 100, and 200 mg/kg BW. The results are expressed as the mean ± SD (*n* = 3). Different letters (a–g) indicate statistically significant differences (*p* < 0.05) among all groups.

PGE levels were measured using ELISA to evaluate the mechanism underlying the anti-inflammatory effects of CXE-NLCs. PGEs are crucial mediators of inflammation^36^. PGE_1_ exerts anti-inflammatory effects that contribute to the suppression of inflammatory responses. Similarly, PGE_2_ is involved in the inflammatory response and promotes various physiological processes associated with inflammation. The PGE_1_-to-PGE_2_ ratio can serve as an indicator of anti-inflammatory effects. Typically, higher levels of PGE_1_ and lower levels of PGE_2_ indicate pronounced anti-inflammatory effects. The effect of CXE-NLCs on carrageenan-induced eicosanoid production is shown in Fig. 6A and **B**. The control (carrageenan only) group showed a significant increase in PGE_1_ and PGE_2_ production. Their expression level in CXE-NLCs significantly decreased in a dose-dependent manner compared to that in the control group. Moreover, L-glutamine (250 mg/kg BW) and CXE-NLCs (200 mg/kg) exhibited similar levels of inhibition, whereas CXE (200 mg/kg BW) showed inhibition similar to that of 100 mg/kg CXE-NLC. Finally, the PGE_1_/PGE_2_ ratio increased by approximately 2-fold in the CXE-NLC groups compared to that in the control group (carrageenan only) (Fig. 6C), indicating enhanced anti-inflammatory effects in vivo.

### Mechanism of action of anti-inflammatory CXE-NLC

**Fig. 7:**
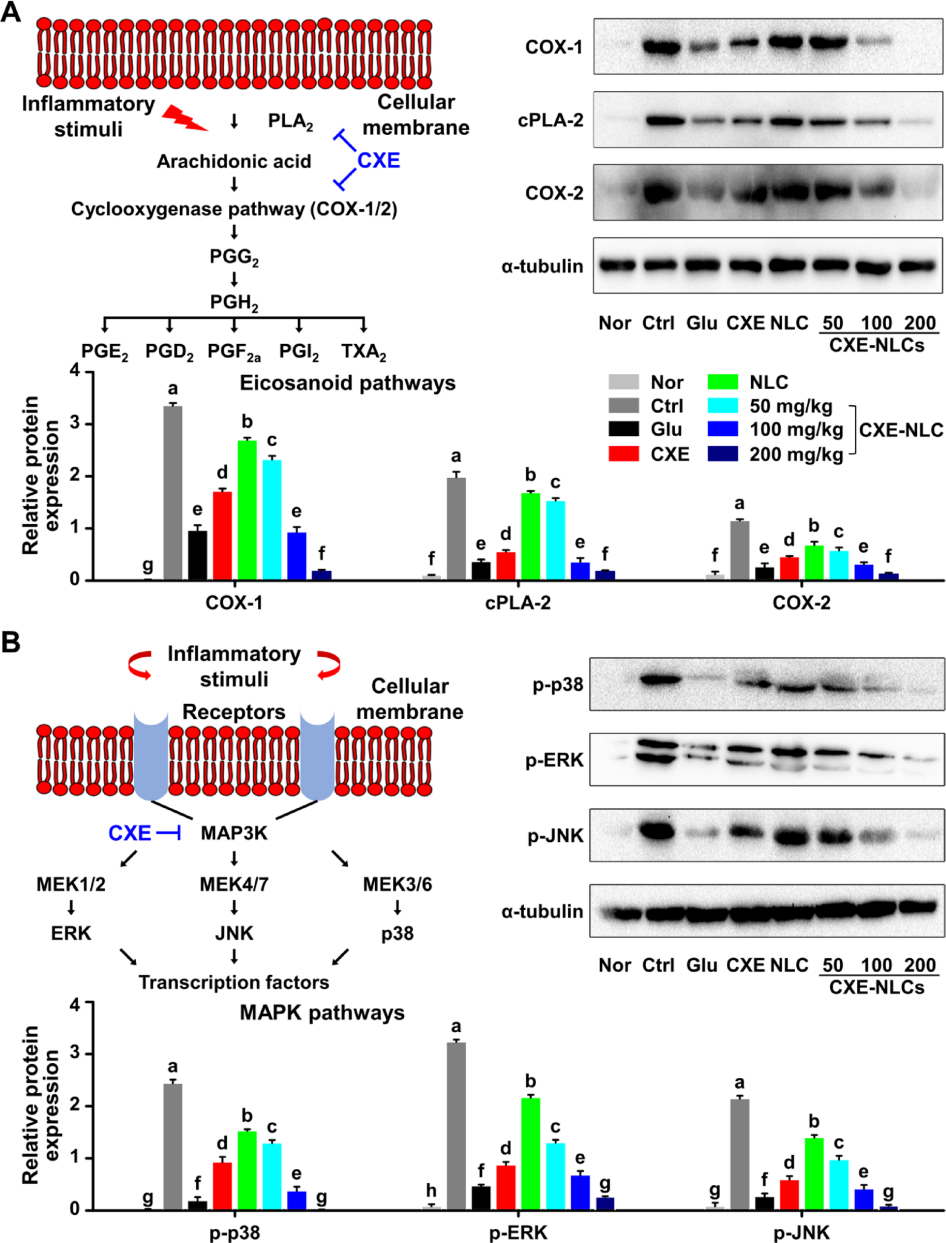
Regulation of CXE-NLC in eicosanoid-associated proteins and MAPK pathways. (**A**) Relative band intensity and western blot analysis of eicosanoids (COXs and cPLA_2_). (**B**) Relative band intensity and western blot analysis of MAPKs (p38, ERK, and JNK). Nor: untreated; Ctrl: carrageenan-treated control; Glu: L-glutamine, 250 mg/kg BW; CXE: free CXE, 200 mg/kg BW; NLC: blank NLC; CXE-NLC: NLCs at dosages of CXE 50, 100, and 200 mg/kg BW. Data are expressed as the mean ± SD (*n* = 3). Different letters (a–g) indicate statistically significant differences (*p* < 0.05) among all groups.

Protein expression in the eicosanoid pathway and phosphorylation levels of the MAPK pathway, which regulate the inflammatory response, were evaluated. Arachidonic acid is converted by cyclooxygenase (COX), an enzyme involved in PG biosynthesis^37^. Commonly used nonsteroidal anti-inflammatory drugs target the COX isoforms COX-1 and COX-2, indicating the involvement of PGs in pain, fever, inflammation, and tumorigenesis^38^. cPLA_2_ contributes to the release of arachidonic acid, and its inhibitors are potential anti-inflammatory drugs. To verify the anti-inflammatory effects of CXE-NLCs on COX and cPLA_2_ pathways, western blotting was performed to determine the expression of COX-1, COX-2, and cPLA_2_. As depicted in Fig. 7A and **Fig. S6**, the control and blank NLC groups exhibited increased expression of COX and cPLA_2_ proteins, whereas CXE-NLCs demonstrated a significant dose-dependent decrease compared to the control group. At a dose of 100 mg/kg, both CXE-NLCs and L-glutamine exhibited similar inhibitory effects on protein expression, whereas the CXE group showed inhibition levels comparable to those of the CXE-NLC 50 or 100 mg/kg groups. Furthermore, 200 mg/kg CXE-NLCs displayed the most pronounced inhibition of COX and cPLA_2_ expression.

MAPK signaling pathways participate in various biological processes, such as cell division, proliferation, and survival^39^. These pathways consist of three branches: extracellular signal-regulated kinase (ERK), c-Jun N-terminal kinase/stress-activated protein kinase (JNK), and p38^39,40^. As shown in Fig. 7B and **Fig. S7**, the control (carrageenan-treated only) and blank NLC groups exhibited increased expression of p38, ERK, and JNK proteins, whereas the CXE-NLC groups showed a significant dose-dependent decrease compared to that in the control group. Similarly, 100 mg/kg CXE-NLCs inhibited protein expression, as observed in the L-glutamine and CXE groups. CXE-NLCs displayed the highest inhibition of p38, ERK, and JNK protein expression, especially at 200 mg/kg. Overall, CXE-NLCs exhibited anti-inflammatory activity in vivo, as evidenced by their effects on the eicosanoid and MAPK pathways.

## Conclusion

In summary, we successfully fabricated and optimized NLC loaded with plant derived extracts as potential orally delivered nanocarriers with anti-inflammatory effects. We validated the performance of this strategy, confirming its effectiveness, not only in physicochemical terms but also in biological functionality. The CXE concentration was optimized based on particle size, zeta potential, colloidal stability, EE, LC, and P_app_. The CXE-NLCs exhibited a nanosize < 200 nm. These nanoparticles demonstrated physiological stability in simulated gastric juice. However, they were gradually degraded in simulated intestinal fluid, ensuring a consistent release profile of the encapsulated bioactive compounds. Encapsulation significantly increased the P_app_ values of the compounds, indicating improved bioaccessibility. The effects of CXE-NLCs on paw size, PGs E1 and E2, the E1/E2 ratio, as well as the eicosanoid and MAPK pathways, further demonstrated their efficient anti-inflammatory potential in vivo.

This approach offers several advantages. (1) CXE-NLCs demonstrated excellent nanoencapsulation efficiency and hydrocolloidal stability, while effectively loading a complex hydrophobic plant derived extract and preserving the bioactivity of its diverse bioactive compounds, such as xanthorrhizol and curcumin. (2) These lipid-based nanoparticles displayed remarkable biostability and sustained-release profiles; they were specifically tailored for oral administration, maintained structural integrity, and controlled release under simulated gastrointestinal conditions. (3) The enhanced dispersity of CXE-NLCs led to improved anti-inflammatory effects in vivo, surpassing those observed with the administration of free CXE; this was likely due to increased oral delivery to target sites. Although these findings are promising, further studies are required to better characterize the pharmacokinetic behavior and immune cell responses of the CXE-NLCs. Additionally, future studies are required to explore other biological activities associated with specific symptoms and diseases, including cancer, and to systematically optimize NLC for various delivery routes beyond oral administration. Overall, this approach holds promise as a versatile oral delivery system to enhance the biological activities of plant derived extracts, making them valuable nutraceuticals and pharmaceuticals.

## Supplementary Information

Below is the link to the electronic supplementary material.

Supplementary Material 1

## Data Availability

Data is provided within the manuscript or supplementary information files.

## Abbreviations

Cur: Curcumin
CXE: *Curcuma xanthorrhiza* extract
CXE-NLC: *Curcuma xanthorrhiza* extract loaded nanostructured lipid carrier
NLC: Nanostructured lipid carriers
RIPA buffer: Radioimmunoprecipitation assay buffer
SGF: Simulated gastric fluid
SIF: Simulated intestinal fluid
SLN: Solid lipid nanoparticle
Xan: Xanthorrhizol

## References

[1] Batra, H., Pawar, S. & Bahl, D. Curcumin in combination with anti-cancer drugs: A nanomedicine review. *Pharmacol. Res.* **139**, 91–105 (2019). 30408575 10.1016/j.phrs.2018.11.005

[2] Fraga, B. M. Natural sesquiterpenoids. *Nat. Prod. Rep.* **30** (9), 1226–1264 (2013). 23884176 10.1039/c3np70047j

[3] Simamora, A. et al. Xanthorrhizol, a potential anticancer agent, from curcuma xanthorrhiza Roxb. *Phytomedicine* **105**, 154359 (2022). 35933899 10.1016/j.phymed.2022.154359

[4] Oon, S. F. et al. Xanthorrhizol: A review of its Pharmacological activities and anticancer properties. *Cancer Cell. Int.* **15** (1), 100 (2015). 26500452 10.1186/s12935-015-0255-4 PMC4618344

[5] Cho, J. Y. et al. Standardized ethanolic extract of the rhizome of curcuma xanthorrhiza prevents murine ulcerative colitis by regulation of inflammation. *J. Funct. Foods*. **30**, 282–289 (2017).

[6] Arifin, M. F. et al. Nanosuspension formula of curcuma Xanthorriza rhizome dry extract: impact of tween 80-PEG 400 ratio. *Sci. Pharm.* **3** (2), 112–119 (2024).

[7] Samran, S. et al. The formulation of dry curcuma (Curcuma xanthorrhiza roxb.) extract microcapsules by spray wet microencapsulation techniques. *Asian J. Pharm. Clin. Res.* **11** (3), 226–229 (2018).

[8] Kim, T. et al. Nanoparticle-patterned multicompartmental Chitosan capsules for oral delivery of oligonucleotides. *ACS Biomater. Sci. Eng.* **4** (12), 4163–4173 (2018). 33418815 10.1021/acsbiomaterials.8b00806

[9] Moradi, M. et al. Interactions between nanoparticle-based food additives and other food ingredients: A review of current knowledge. *Trends Food Sci. Technol.* **120**, 75–87 (2022).

[10] Yang, K. et al. Mucoadhesive Chitosan microcapsules for controlled Gastrointestinal delivery and oral bioavailability enhancement of low molecular weight peptides. *J. Control Release*. **365**, 422–434 (2024). 37863357 10.1016/j.jconrel.2023.10.021

[11] Müller, R. H., Radtke, M. & Wissing, S. A. Solid lipid nanoparticles (SLN) and nanostructured lipid carriers (NLC) in cosmetic and dermatological preparations. *Adv. Drug Deliv Rev.* **54**, S131–S155 (2002). 12460720 10.1016/s0169-409x(02)00118-7

[12] Katouzian, I. et al. Formulation and application of a new generation of lipid nano-carriers for the food bioactive ingredients. *Trends Food Sci. Technol.* **68**, 14–25 (2017).

[13] Park, S. J. et al. Improvement of curcuminoid bioaccessibility from turmeric by a nanostructured lipid carrier system. *Food Chem.* **251**, 51–57 (2018). 29426423 10.1016/j.foodchem.2018.01.071

[14] Tenchov, R. et al. Lipid nanoparticles from liposomes to mRNA vaccine delivery, a landscape of research diversity and advancement. *ACS Nano*. **15** (11), 16982–17015 (2021). 34181394 10.1021/acsnano.1c04996

[15] Nathan, C. Points of control in inflammation. *Nature* **420** (6917), 846–852 (2002). 12490957 10.1038/nature01320

[16] Lee, J. et al. Protective effect of tremella fuciformis Berk extract on LPS-induced acute inflammation via Inhibition of the NF-κB and MAPK pathways. *Food Funct.* **7** (7), 3263–3272 (2016). 27334265 10.1039/c6fo00540c

[17] Li, Z. et al. Xuanfei Baidu formula alleviates impaired mitochondrial dynamics and activated NLRP3 inflammasome by repressing NF-κB and MAPK pathways in LPS-induced ALI and inflammation models. *Phytomedicine* **108**, 154545 (2023). 36423572 10.1016/j.phymed.2022.154545 PMC9643338

[18] Cho, J. Y., Hwang, J. K. & Chun, H. S. Xanthorrhizol attenuates dextran sulfate sodium-induced colitis via the modulation of the expression of inflammatory genes in mice. *Life Sci.* **88** (19–20), 864–870 (2011). 21419136 10.1016/j.lfs.2011.03.007

[19] Chung, W. Y. et al. Xanthorrhizol inhibits 12-O-tetradecanoylphorbol-13-acetate-induced acute inflammation and two-stage mouse skin carcinogenesis by blocking the expression of ornithine decarboxylase, cyclooxygenase-2 and inducible nitric oxide synthase through mitogen-activated protein kinases and/or the nuclear factor-κB. *Carcinogenesis* **28** (6), 1224–1231 (2007). 17234720 10.1093/carcin/bgm005

[20] Zhou, M. et al. Xanthorrhizol ameliorates oxidative stress and inflammation in freund’s complete adjuvant-induced rheumatoid arthritis in rats. *Appl. Biochem. Biotechnol.* **194** (12), 6423–6437 (2022). 35932370 10.1007/s12010-022-04091-4

[21] Ramasamy, T. et al. Layer-by-layer coated lipid–polymer hybrid nanoparticles designed for use in anticancer drug delivery. *Carbohydr. Polym.* **102**, 653–661 (2014). 24507332 10.1016/j.carbpol.2013.11.009

[22] Aditya, N. P. et al. Curcumin and genistein coloaded nanostructured lipid carriers: in vitro digestion and antiprostate cancer activity. *J. Agric. Food Chem.* **61** (8), 1878–1883 (2013). 23362941 10.1021/jf305143k

[23] Hyun, J. E. et al. Digestion stability of curcumin-loaded nanostructured lipid carrier. *LWT* **162**, 113474 (2022).

[24] Araiza-Calahorra, A., Akhtar, M. & Sarkar, A. Recent advances in emulsion-based delivery approaches for curcumin: from encapsulation to bioaccessibility. *Trends Food Sci. Technol.* **71**, 155–169 (2018).

[25] Wang, K. et al. Enhancement of oral bioavailability of cyclosporine A: comparison of various nanoscale drug-delivery systems. *Int. J. Nanomed.* **9**, 4991–4999 (2014). 10.2147/IJN.S72560 PMC4218918 25378925

[26] Weber, J. et al. Oxidation of polysorbates–An underestimated degradation pathway? *Int. J. Pharm. : X*. **6**, 100202 (2023). 37680877 10.1016/j.ijpx.2023.100202 PMC10480556

[27] Kim, J. U. et al. Optimization of phytic acid-crosslinked Chitosan microspheres for oral insulin delivery using response surface methodology. *Int. J. Pharm.* **588**, 119736 (2020). 32758596 10.1016/j.ijpharm.2020.119736

[28] Zhang, Q. et al. Nanostructured lipid carriers with exceptional Gastrointestinal stability and Inhibition of P-gp efflux for improved oral delivery of Tilmicosin. *Colloids Surf. B Biointerfaces*. **187**, 110649 (2020). 31767412 10.1016/j.colsurfb.2019.110649

[29] Rohaeti, E. et al. Fourier transform infrared spectroscopy combined with chemometrics for discrimination of curcuma longa, curcuma xanthorrhiza and Zingiber cassumunar. *Spectrochim Acta Mol. Biomol. Spectrosc.* **137**, 1244–1249 (2015). 10.1016/j.saa.2014.08.139 25305617

[30] Izza, N. M. et al. Dependence of the core–shell structure on the lipid composition of nanostructured lipid carriers: implications for drug carrier design. *ACS Appl. Nano Mater.* **5** (7), 9958–9969 (2022).

[31] Aditya, N. P. et al. Development and evaluation of lipid nanocarriers for Quercetin delivery: A comparative study of solid lipid nanoparticles (SLN), nanostructured lipid carriers (NLC), and lipid nanoemulsions (LNE). *LWT* **59** (1), 115–121 (2014).

[32] Gleeson, J. P., Ryan, S. M. & Brayden, D. J. Oral delivery strategies for nutraceuticals: delivery vehicles and absorption enhancers. *Trends Food Sci. Technol.* **53**, 90–101 (2016).

[33] Soleimanian, Y. et al. β-Sitosterol loaded nanostructured lipid carrier: physical and oxidative stability, in vitro simulated digestion and hypocholesterolemic activity. *Pharmaceutics* **12** (4), 386 (2020). 32331384 10.3390/pharmaceutics12040386 PMC7237988

[34] Lee, S. G. et al. RIPL peptide-conjugated nanostructured lipid carriers for enhanced intracellular drug delivery to hepsin-expressing cancer cells. *Int. J. Nanomed.* **13**, 3263–3278 (2018). 10.2147/IJN.S166021 PMC5987859 29910614

[35] Elena, T. et al. Anti-inflammatory effects of adrenomedullin on acute lung injury induced by Carrageenan in mice. *Mediators Inflamm.* 717851 (2012). (2012) (1). 10.1155/2012/717851 PMC3364017 22685374

[36] Bamba, H. et al. Effect of rebamipide on prostaglandin receptors-mediated increase of inflammatory cytokine production by macrophages. *Aliment. Pharmacol. Ther.* **18**, 113–118 (2003). 12925148 10.1046/j.1365-2036.18.s1.13.x

[37] Stables, M. J. & Gilroy, D. W. Old and new generation lipid mediators in acute inflammation and resolution. *Prog Lipid Res.* **50** (1), 35–51 (2011). 20655950 10.1016/j.plipres.2010.07.005

[38] Nichols, K. M. et al. A consolidated linkage map for rainbow trout (Oncorhynchus mykiss). *Anim. Genet.* **34** (2), 102–115 (2003). 12648093 10.1046/j.1365-2052.2003.00957.x

[39] Thalhamer, T., McGrath, M. A. & Harnett, M. M. MAPKs and their relevance to arthritis and inflammation. *Rheumatology* **47** (4), 409–414 (2008). 18187523 10.1093/rheumatology/kem297

[40] Monmai, C. et al. Anti-inflammatory effect of Asterias amurensis fatty acids through NF-κB and MAPK pathways against LPS-stimulated RAW264. 7 cells. *J. Microbiol. Biotechnol.* **28** (10), 1635–1644 (2018). 30441883 10.4014/jmb.1802.03044

