# Supplementary material for: Plant derived extract loaded nanostructured lipid carriers with enhanced oral delivery for the treatment of acute inflammation
Source: Sci Rep. 2025 Oct 3;15:34551. doi: 10.1038/s41598-025-17898-y (PMC12494995; doi:10.1038/s41598-025-17898-y)
Supplement: Supplementary file 1 — Supplementary Material 1 [file 41598_2025_17898_MOESM1_ESM.pdf]

Supplementary Materials

Plant derived extract loaded nanostructured lipid carriers with enhanced oral delivery for the treatment of acute inflammation

Eun Yong Lee[a,†], JeongUn Choi[b,†], Young Min Kim[c], Kyungjik Yang[c], A-yeong Jang[b], Hae Kyung Shin[c] Hae Rim Kim[a], Keonwook Nam[c], Jae Kwan Hwang[c], Jiyong Park[d], Woo Jung Park[b,e,*], Young Hoon Roh[a,c,*]

[a] Graduate Program in Bioindustrial Engineering, College of Life Science and Biotechnology, Yonsei University, 50 Yonsei-ro, Seodaemun-gu, Seoul 03722, Republic of Korea

[b] Department of Marine Food Science and Technology, Gangneung-Wonju National University, Gangneung 25457, Republic of Korea

[c] Department of Biotechnology, College of Life Science and Biotechnology, Yonsei University, 50 Yonsei-ro, Seodaemun-gu, Seoul 03722, Republic of Korea

[d] Nutrex Technology, 43 Changeop-ro A-801, Seongnam 13449, Republic of Korea

[e] Department of Marine Bio Food Science, Gangneung-Wonju National University, Gangneung 25457, Republic of Korea

[*]Address correspondence to:

Woo Jung Park, Ph.D., Email: pwj0505@gwnu.ac.kr

Young Hoon Roh. Ph.D., Email: yr36@yonsei.ac.kr

[†]These authors contributed equally to this work.

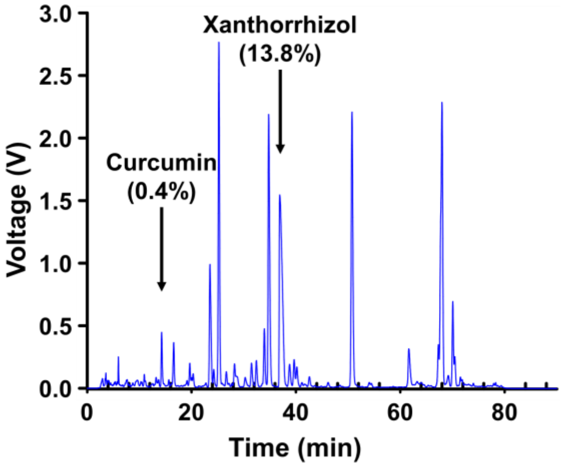

Fig. S1. HPLC chromatogram of CXE. CXE is mixture of various curcuminoids and other phytochemicals. The elution time and contents of curcumin and xanthorrhizol is marked by arrow.

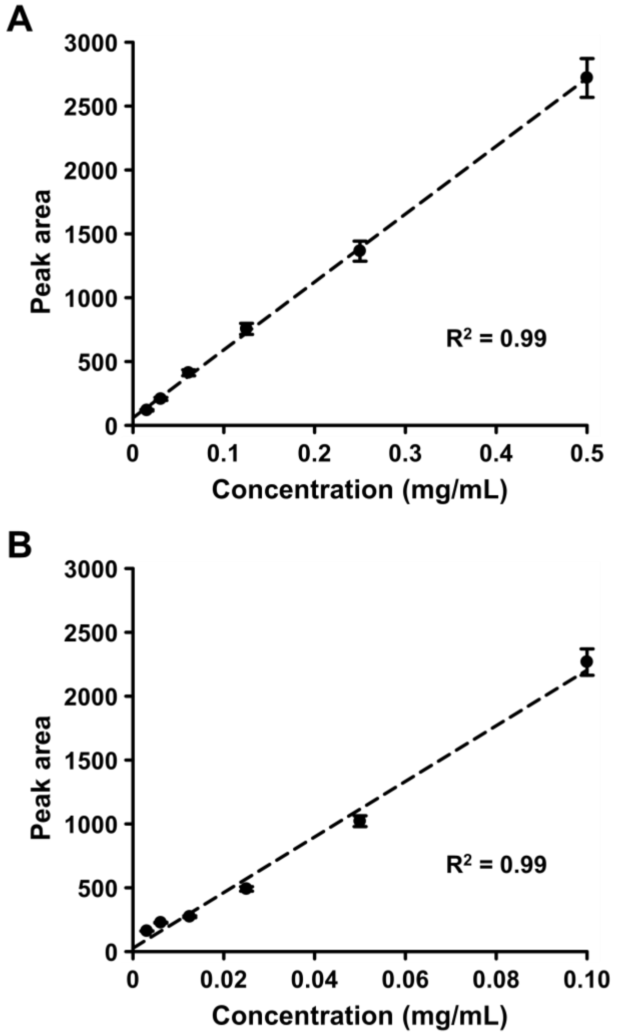

Fig. S2. Standard curve of curcumin and xanthorrhizol measured by HPLC. (A) A standard calibration curve for xanthorrhizol was prepared using the mean peak area as a function of the concentration, ranging from 0.016 to 0.5 mg/ml. (B) A standard calibration curve for curcumin was prepared using the mean peak area as a function of the concentration, ranging from 0.003 to 0.1 mg/ml.

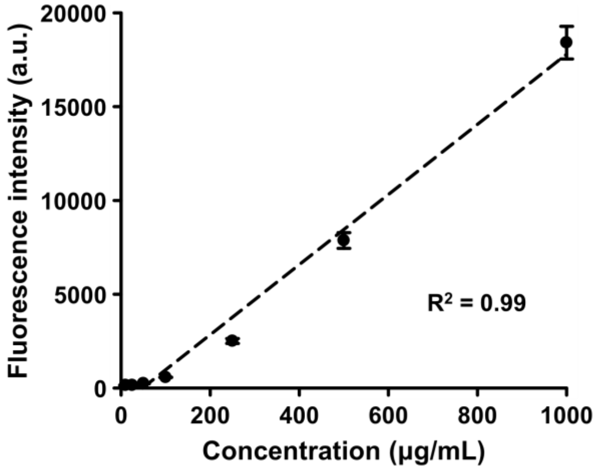

Fig. S3. Standard curve of *Curcuma xanthorrhiza* extract measured by fluorescence. A standard calibration curve for CXE was prepared using the fluorescence intensity as a function of the concentration, ranging from 0 to 1000 µg/ml

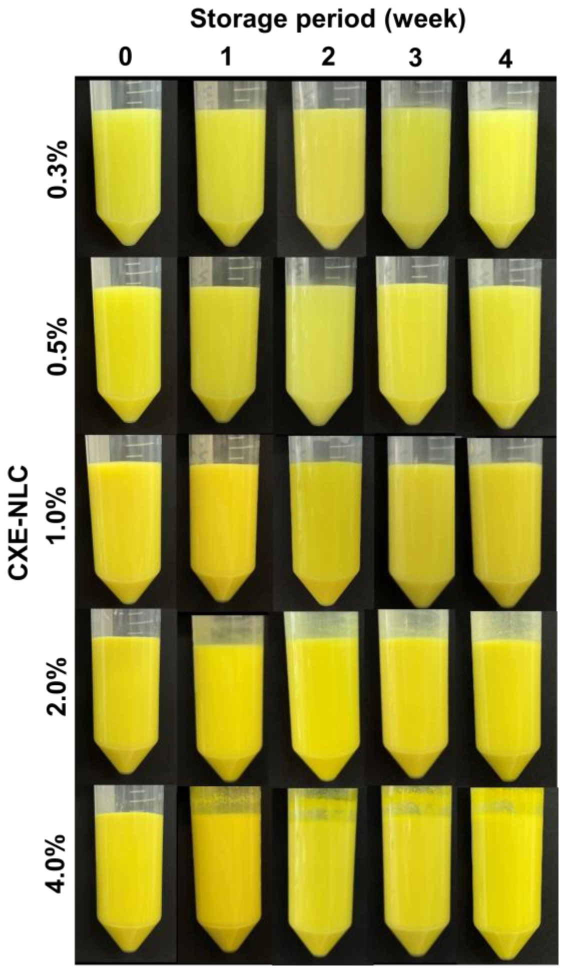

Fig. S4. Colloidal stability of CXE-NLCs over four weeks of storage. The colloidal stability of CXE-NLCs with varying CXE concentrations (0.3–4.0%, w/v) under refrigerated conditions (4 °C) was observed by digital images.

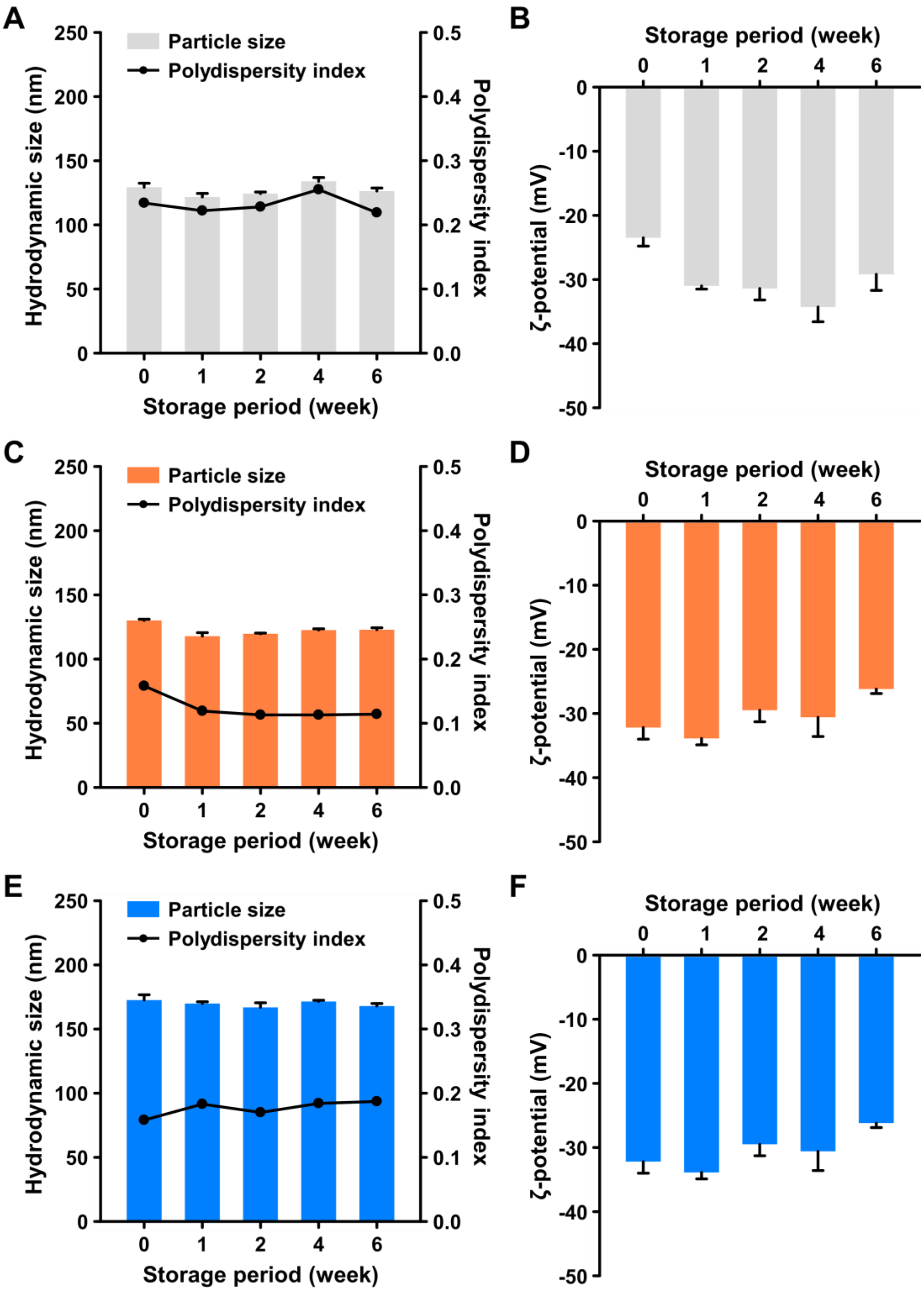

Fig. S5. Hydrodynamic diameter, polydispersity index, and zeta potential of CXE-NLC at 4 °C during six weeks of storage. (A, B) CXE-NLC-0.3. (C, D) CXE-NLC-0.5. (E, F) CXE-NLC-1.0.

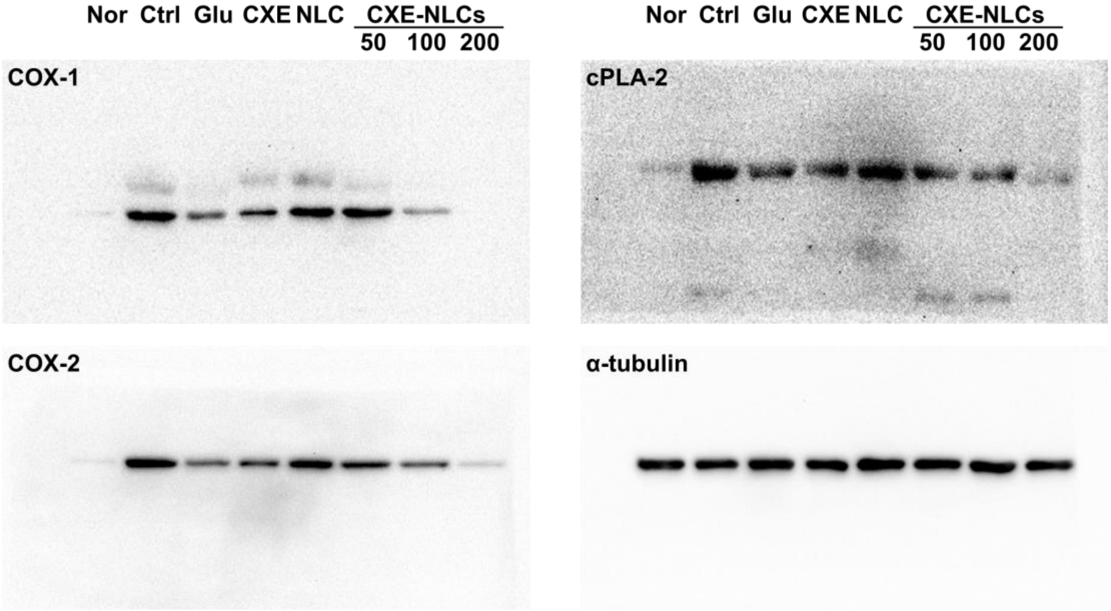

Fig. S6. Western blot analysis for eicosanoid pathway associated proteins. The representative western blot analysis images of COX-1, cPLA-2, COX-2, and α-tubulin. Nor: untreated. Ctrl: control as carrageenan-treated only. Glu: L-glutamine 250 mg/kg BW. CXE: CXE 200 mg/kg BW. NLC: blank NLC. CXE-NLC: NLCs at dosages of CXE 50, 100, and 200 mg/kg BW.

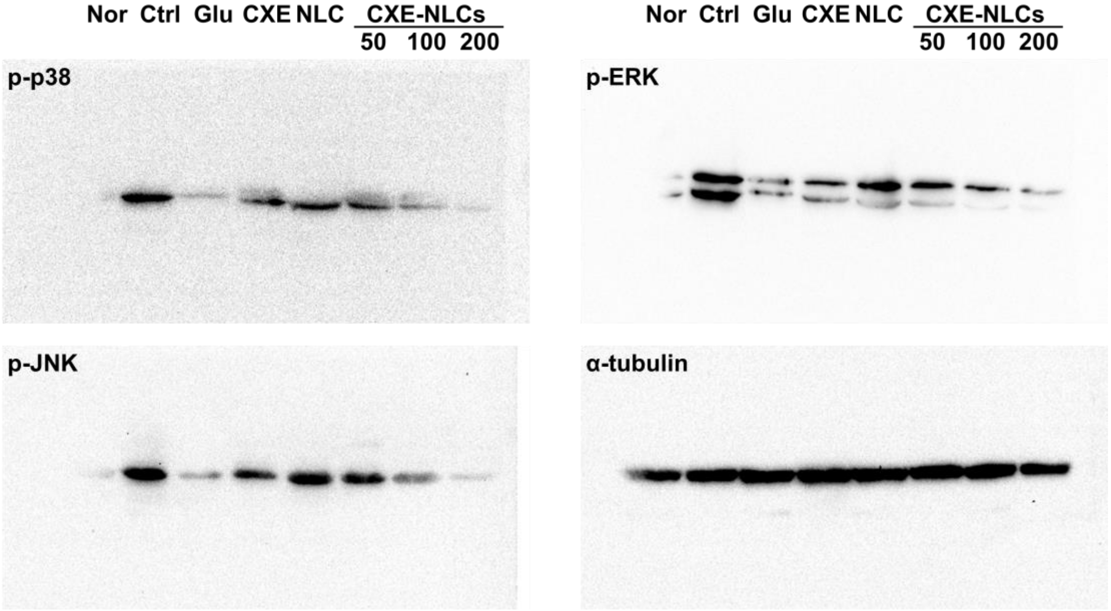

Fig. S7. Western blot analysis for MAPKs pathway associated proteins. The representative western blot analysis images of p38, ERK, JNK, and α-tubulin. Nor: untreated. Ctrl: control as carrageenan-treated only. Glu: L-glutamine 250 mg/kg BW. CXE: CXE 200 mg/kg BW. NLC: blank NLC. CXE-NLC: NLCs at dosages of CXE 50, 100, and 200 mg/kg BW.

Table S1. Hydrodynamic diameter, polydispersity index, and zeta potential of CXE-NLCs at 4 °C during six weeks of storage.

Week	Type	Size (nm)	PDI	ζ-Potential (mV)
**0**	CXE-NLC-0.3	129.3 ± 3.1	0.234	-23.5 ± 1.3
	CXE-NLC-0.5	130.0 ± 1.0	0.103	-32.2 ± 1.8
	CXE-NLC-1.0	172.6 ± 4.1	0.158	-24.9 ± 0.9
	CXE-NLC-2.0	241.9 ± 5.9	0.254	-32.0 ± 0.4
	CXE-NLC-4.0	277.3 ± 4.0	0.270	-30.4 ± 1.7
**1**	CXE-NLC-0.3	121.7 ± 2.8	0.222	-31.0 ± 0.5
	CXE-NLC-0.5	117.8 ± 2.7	0.119	-33.9 ± 1.0
	CXE-NLC-1.0	169.9 ± 1.3	0.183	-33.2 ± 3.2
	CXE-NLC-2.0	249.9 ± 5.9	0.237	-37.2 ± 2.1
	CXE-NLC-4.0	N/A	N/A	N/A
**2**	CXE-NLC-0.3	124.3 ± 1.3	0.228	-31.4 ± 1.8
	CXE-NLC-0.5	119.6 ± 0.6	0.113	-29.5 ± 1.8
	CXE-NLC-1.0	166.9 ± 3.5	0.170	-29.9 ± 0.3
	CXE-NLC-2.0	N/A	N/A	N/A
	CXE-NLC-4.0	N/A	N/A	N/A
**4**	CXE-NLC-0.3	133.9 ± 3.0	0.255	-34.3 ± 2.3
	CXE-NLC-0.5	122.6 ± 0.9	0.113	-30.6 ± 3.0
	CXE-NLC-1.0	171.5 ± 0.9	0.184	-33.5 ± 0.5
	CXE-NLC-2.0	N/A	N/A	N/A
	CXE-NLC-4.0	N/A	N/A	N/A
**6**	CXE-NLC-0.3	126.3 ± 2.4	0.219	-29.2 ± 2.5
	CXE-NLC-0.5	122.8 ± 1.5	0.114	-26.2 ± 0.7
	CXE-NLC-1.0	167.9 ± 2.0	0.187	-24.2 ± 3.0
	CXE-NLC-2.0	N/A	N/A	N/A
	CXE-NLC-4.0	N/A	N/A	N/A

(N/A; not applicable)
